# Adolescent idiopathic scoliosis (AIS) treated with arthrodesis and posterior titanium instrumentation: 8 to 12 years follow up without late infection

**DOI:** 10.1186/1748-7161-4-16

**Published:** 2009-08-12

**Authors:** Franz J Mueller, Herbert Gluch

**Affiliations:** 1 Department of Spinal Surgery and Scoliosis Center, Behandlungszentrum Vogtareuth, Germany

## Abstract

**Background:**

There are no data in the peer-reviewed literature regarding long term results in patients treated for AIS with a posterior titanium instrumentation. Therefore we assessed the outcome in 50 patients treated by titanium implant.

**Methods:**

A total of 50 patients with a mean age of 16.6 years were treated. In all patients, titanium hooks and pedicle screws were used in combination. The demographic data and the pre- and post-operative radiographs of all 50 patients were re-examined, and 49 of the 50 patients (98%) attended a radiological and clinical follow up-examination on average 10.1 years post-operatively. The clinical results were recorded by means of the SRS 24 questionnaire.

**Results:**

In the frontal plane, the mean pre-operative thoracic and lumbar curve had been 62.4° and 43.5° respectively, post-operatively the curves were reduced to 26.9° and 16.3°, resulting in a correction rate of 56.9% for thoracic and 62.5% for lumbar curve. At the follow up-evaluation, the Cobb angle of the thoracic and lumbar curve was 31.0° and 21.3° respectively, giving a final correction rate of 50.3% for thoracic, and 51.0% for lumbar curve. 7 of the 50 patients (14.3%) had undergo revision surgery for complications, but complete implant removal was necessary in only one case. Analysis of the SRS 24 questionnaire showed an average score of 95.8 points.

**Conclusion:**

Posterior titanium instrumentation is a safe and effective procedure in the surgical correction of AIS. In this retrospective study with small patient number, it shows favourable long-term results; in particular, the loss of correction is low, no late infection occurred and there was a very high survival rate of the implant itself.

## Introduction

Various anterior and posterior operative procedures are available for the treatment of adolescent idiopathic scoliosis (AIS). Among the posterior procedures, two different systems can be distinguished: With the development of the Harrington rod a long segment implant first became available and has established itself as the most frequently applied method for the operative treatment of the various forms of scoliosis [1]. With this system, frontal correction is achieved primarily by distraction of the spine with elongation of the concave side of the curve. The procedure also requires post-operative stabilization of the trunk with an orthesis or plaster cast for several months in order to maintain the achieved correction. These disadvantages led to the development of the Cotrel-Dubousset (CD) double-rod technique [2], which achieves not only better sagittal and frontal correction but also avoids post-operative immobilization due to greater primary stability [3]. Numerous modifications of the CD system followed [4,5]; besides segmental hooks and sublaminar wires, transpedicle screws are now

also increasingly used for correction because they can improve the primary stability [6]. In our department a double-rod system made of titanium has been used exclusively since 1993 for the operative correction of scoliosis which allows correction through segmental hooks and/or transpedicle screws by "over the top" loading (System WSI Titan, Peter Brehm Chirurgie Mechanik, 91084 Weisendorf, Germany). The aim of our study was therefore to examine the long term efficacy and safety of this titanium implant in AIS.

## Materials and methods

### Patients

Between January 1993 and March 1996 a total of 50 patients with AIS underwent operative correction with the titanium implant. The study sample consisted of 44 females and 6 males with an average age of 16 years (range 12 to 21 years) at the time of surgery. According to the SRS terminology there were 30 thoracic, 16 double, 3 thoracolumbar and 1 lumbar curves. According to the King classification [7] there were 2 patients with Type I, 25 patients with Type II, 9 patients with Type III, 7 patients with Type IV, and 3 patients with Type V curves. 4 patients could not be classified according to King (3× thoracolumbar, 1× left lumbar).

### Surgical procedure

**Figure 1:**
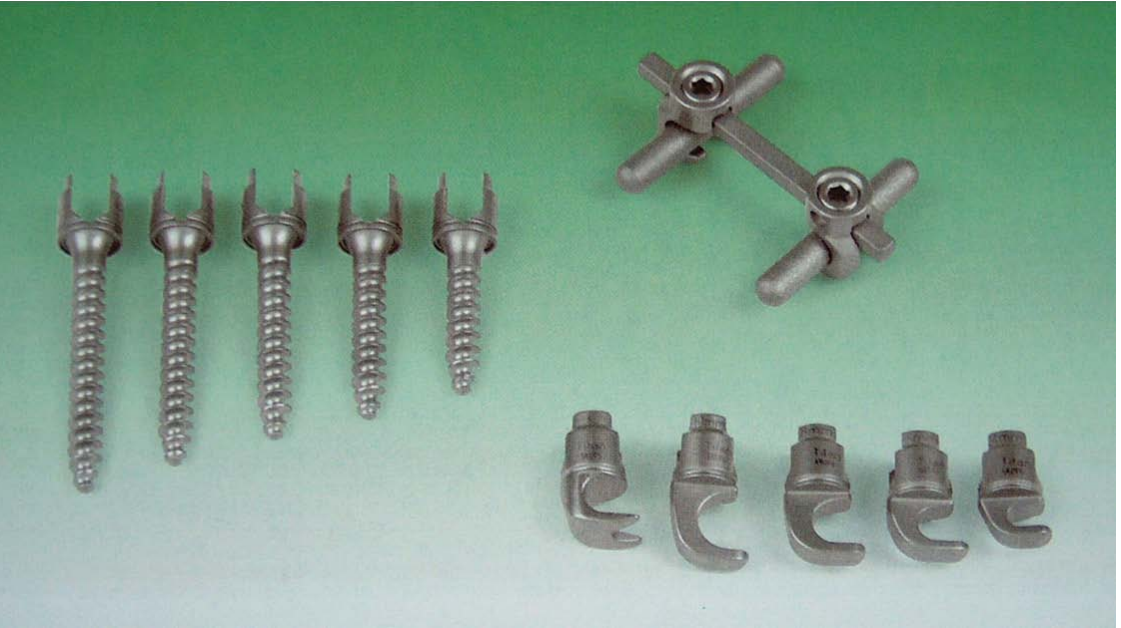
**Instrumentation system WSI Titan, an over the top loading system with screws and hooks**.

The implant is a double rod system made of pure titanium rods with a diameter of 6 or 7 mm, respectively, which allows segmental fixation through lamina hooks and/or conical pedicle screws fabricated from TiAl6V4 alloy. It is a "over the top" loading system (Figure 1). The indication for operation was progression of the AIS with a main curve of over 45° in the frontal plane. Anterior release to mobilize a rigid main curve was not an exclusion criterion. No additional procedures such as rib osteotomy were performed in any of the patients. For preparation and mobilization for the operation, all patients carried out Cotrel self-extension over a period of 2 to 3 weeks. All patients received a perioperative antibiotic prophylaxis with intravenous cephalosporin. All operative procedures were performed by the senior author (H.G.). The operative procedure involved mobilization of the scoliosis by resection of the spinous processes, decortication of the laminae, facet joint cleaning and division of the ligamentum flavum on the concave side of the curvature. The scoliosis was then corrected by inserting the hooks and pedicle screws with loading of the two anatomically shaped vertical rods with rotation in situ and additional compression or distraction of segments as needed. The fifth lumbar vertebra or the sacrum were spared from fusion in all cases. A transverse connector was placed between the cranial and caudal ends of the two vertical rods, respectively, in all patients. A wake up test was performed after insertion of the vertical rod on the concave side to assess the neurological function intra-operatively. For spondylodesis only local bone material reduced to chips was utilized. All patients received autologous blood with or without cell saver, no patient received non- autologous blood products. All patients were mobilized routinely without a corset. Instrumentation with the titanium implant was performed in all cases. Additionally, 9 of 50 patients had anterior release pre-operatively because of a severe main thoracic curve (average 81°, range 71° to 111°).

### Baseline evaluation

Initially the demographic data of all 50 patients was recorded from the medical files. From pre- and post-operative spinal radiographs in both planes (cassettes with 36 inches length) the following parameters were assessed: frontal main and secondary curves using the Cobb method [8]; sagittal thoracic kyphosis angle measured from T5 to T12 and lordosis angle from L1 to S1, also using the Cobb method; frontal balance, determined on the basis of horizontal distance from the center of the C7 vertebral body to the center of the sacrum; apical vertebral translation (distance between the plumbline and the mid portion of the vertebral body at the apex of the 7 curve). Attention was also paid to radiological complications such as rod fracture, pedicle screw fracture or hook dislocation.

### Follow up-evaluation

Following evaluation of the demographic and radiological data between January 2005 and August 2005, the patients were invited by telephone to attend a follow up- examination. The mean follow up was 121.7 ± 14.1 months (range 101 to 151 months) respectively 10.1 years. 49 of the 50 patients (98%) accepted the invitation to follow up; only one patient had moved abroad and could not be contacted for the questionnaire, but the demographic data and the pre- and post-operative radiological results and those at the time of the last follow up were included. At follow up, the radiological examinations mentioned above were repeated and compared with the previous films. The patients were also given a questionnaire of the Scoliosis Research Society with 24 questions, which measures the quality of life of scoliosis patients. The SRS 24 questionnaire is a disease-specific, reliable and validated questionnaire used to assess outcomes in AIS [9]. The questionnaire includes 24 questions, and the maximum possible score is 120 points, indicating that the patient is highly satisfied and asymptomatic.

### Statistical analysis

SPSS Version 8 software for Window was used for the statistical analysis, part for frontal and sagittal balances of all patients pre-, postoperative and at final follow-up time and p values of ≤ 0.05 were considered significant. Descriptive statistics were used to determine means, standard deviations (SD) and ranges. Comparisons between variables were performed using Student's t test and the Kruskal-Wallis test.

## Results

The blood loss was 1980 ml on average (range 600 to 4500 ml), and the operation time was 270 min on average (range 140 to 410 min). Fusion with the implant included 10.0 (range 6 to 13) vertebrae on average. The average cephalic level of fusion was T 5.2 and the average caudal level of fusion was L 2.3. The two rods were fixed with a combination of hooks and pedicle screws only, with an average of 8.9 hooks and 4.2 screws per patient. A total of 424 hooks and 209 pedicle screws were inserted in 50 patients. The pedicle screws were inserted mainly in the lumbar region of the instrumentation.

**Figure 2:**
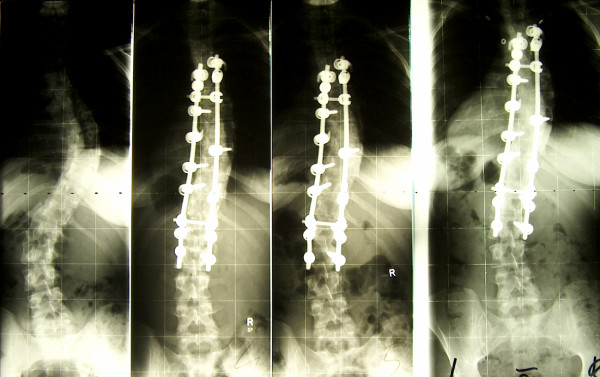
**2A – 2D female patients; 15 years old at time of surgery; Curve: King 2, Instrumentation with titanium implant T5 to L2; SRS 24 questionaire at follow up: 98 of max. 120 points**. 2A: Cobb preop: thoracic 61°, lumbal 46°, 2B: Cobb postop: thoracic 34°, lumbal 20° 2C: Cobb 15 months postop: thoracic 34°, lumbal 32° 2D: Cobb 101 months postop: thoracic 34°, lumbal 38°

The results of frontal and sagittal plane radiography at baseline, post-operatively and at the most recent follow up are shown at table 1. There was no direct or indirect operative mortality, and there were also no permanent neurological complications. According to the medical files, 7 revisions had to be performed (Table 2): Re-instrumentation had to be performed in 2 patients because of radiological evidence of pseudarthrosis with loosening of the implant and loss of correction. In these two patients the implant is still in situ at the time of follow up. Complete implant removal was undertaken in only one patient because of persistent back pain in the region of the instrumentation (late operative site pain). Intra-operatively there was slight metallosis and there was no evidence of infection or pseudarthrosis. We also recorded the following complications, which did not require revision: 8 patients had an asymptomatic hook dislocation, in each case in the distal region of the instrumentation. In 2 further patients there was dislocation of the vertical rod and in 4 further patients the last radiological follow up showed an asymptomatic fracture of the vertical rod without significant distraction of the fracture site as possible evidence of pseudarthrosis. There was no case of pedicle screw fatigue fracture or fracture of a transverse connector rod. The cumulative rate of re-operation was 14.0%. It must be emphasized that we did not see a late deep infection in any patient, and only one implant had been completely removed at the time of follow up. In the SRS questionnaire, the total score averaged 95.8 ± 8.8 points out of maximum of 120 points (range 60 – 110 points) at the final follow up. In the questionnaire, 48 of 49 patients (98%) were highly or fairly satisfied with the result of the back treatment; only one female patient was somewhat dissatisfied – however we saw no objective (e.g. loss of correction) signs concerning this result. 42 of 49 patients (86%) reported to suffer never or rarely from back pain at rest. Overall 44 of 49 patients (90%) would definitely or probably undergo the same treatment again. The SRS scores did not depend significantly (p > 0.05) on the scoliosis classification or gender, either (Figure 2A–2D).

**Table 1:** Results of frontal and sagittal plane radiography at baseline, post-operatively and at the most recent follow up (mean ± standard deviation)

	baseline	postop	correction rate	follow up	correction rate
Thoracic curve (°)	62.4 ± 14.1	26.9 ± 9.8	56.9%	31.0 ± 11.2	50.3%
Lumbar curve (°)	43.5 ± 14.9	16.3 ± 9.9	62.5%	21.3 ± 13.3	51.0%
Apical translation (mm)	51.7 ± 26.2	18.7 ± 14.7	63.8%	25.3 ± 11.9	51.1%
Frontal balance (mm)	15.4 ± 12.4	13.8 ± 1.5		10.8 ± 9.7	
Thoracic kyphosis angle curve (°)	19.1 ± 14.5	22.1 ± 11.1		25.8 ± 12.3	
Lumbar lordosis angle curve (°)	56.1 ± 12.8	55.9 ± 10.2		57.9 ± 9.9	

**Table 2:** Overall reoperation rate for WSI titanium instrumentation

complications	number of patients	operative revision after...
Neurologic complication	1	1 day
acute infection	1	3 weeks
implant failure	1	3 weeks
loss of correction	1	22 months
late operative site pain (LOSP)	1	43 months
pseudarthrosis	2	73 and 102 months
late deep infection	0	-
reoperation for all reasons	7/50 (14%)	

## Discussion

Surgical correction of AIS in general can be regarded as a safe and proven procedure yielding favorable long-term results superior to those of a brace treatment [10]. However, the existing and evolving multitude of methods and systems [11,12] underlines that there is no established and internationally accepted standard for the surgical procedure. Although the Harrington rod (developed originally for patients with poliomyelitis) was used most frequently throughout the world for operative correction of AIS for over 40 years, overall long-term results are scarcely examined and often refer only to specific questions [13], This might be due to the fact that many of these implants were removed after only a few years, routinely or because of complications [14]. For the implants with pedicle screws and hooks fixation very few long-term results have been published too, although these systems has been available as an implant for over 20 years: Helenius et al. [15] investigated 57 patients with AIS and CD instrumentation a mean of 13.0 years post-operatively. Pre-operatively the average frontal thoracic Cobb angle had been 55°. Upon follow up, the average thoracic curve was 32°, resulting in a 42% correction. The frontal lumbar curve showed an average correction of 32% at the time of follow up. The documented complications were one case of acute (1.7%) and three cases (5.3%) of late deep infection, but prophylactic antibiotics (single shot) had not been administered. Clinically, the average SRS score was 97 points, and 6 patients (10.5%) reported back pain often or very often. Cook et al. [16] investigated 49 patients a mean of 105 months post-operatively with the isolated question of the revision rate. 12 of 49 patients (24.5%) had to undergo revision surgery. The indications were late operative site pain (LOSP) (12.2%), prominence or improperly placed implant, pseudarthrosis and late deep infection. Clark and Shufflebarger [17] investigated 917 patients with a CD implant retrospectively for late deep infection. A total of 21 patients (2.3%) were documented. In all cases complete implant removal was performed. The initial symptom had been development of a fistula or a fluctuant swelling below the skin an average of 3.1 years post-operatively. Bago et al. [18] presented a survivorship analysis of CD instrumentation in the surgical treatment of idiopathic scoliosis. Re-operation, usually implant removal, had to be performed in 23 of 113 patients (20.3%). The reason for the majority of re-operations was late infection, followed by mechanical failure, LOSP and acute infection. We also have had observed a much higher revision rate with CD implants previously used in our department before the introduction of titanium 1993 [19]. Hahn et al. [20] showed an incidence of late infection of 7.5% for AIS, treated with universal spine system (USS). Remes et al. [21] reported 3 out of 55 patients (7.3%) with late deep infection after correction of AIS with USS, and Richards [5] reported 10 patients (6.7%) who had late drainage at a mean of 25 months after placement of Texas Scottish Rite Hospital (TSRH) instrumentation to correct AIS. Therefore, the titanium implant in this study seems to be favorable in particular with respect to loss of correction, revision surgery for late deep infection or LOSP. The cause of LOSP has not so far been confirmed; mechanical irritation caused by the implant itself is suggested in particular, and also pseudarthrosis, implant loosening, bursa formation or subclinical infections. In our study, late deep infection was not observed in any of the patients, which is in contrast to most of the published series with late deep infection rates typically between 2% and 9% (Table 3). The reasons for this difference, should it be systematic, remain unclear, because the systems differ both in material and design. However, titanium alloy itself is corrosion-resistant, biocompatible and bioadhesive in vitro and in vivo and demonstrates a markedly low adherence for bacteria [22-24].

**Table 3:** Reoperation rates for late infection: Comparison of published results

Authors	implant system	Patients	reoperation rate
Richards S. [5]	TSHR	10 out of 149	6.7%
Clark CE. [17]	CD	21 out of 917	2.3%
Helenius I. [15]	CD	3 out of 57	5.3%
Bago J. [18]	CD	10 out of 113	8.8%
Remes V. [21]	CD	3 out of 57	5.3%
Remes V. [21]	USS	4 out of 55	7.3%
Hahn F. [20]	USS	5 out of 67	7.5%

## Conclusion

In summery, the study presents the first long- term results with a posterior titanium instrumentation for the treatment of adolescent idiopathic scoliosis. The results are encouraging: the implant is safe and effective with a high level of patients satisfaction; in particular, no late infection occurred and there was a very high survival rate of the implant itself. However the study design was retrospective and the patient number small.

## Competing interests

The authors declare that they have no competing interests.

## Authors' contributions

FJM has contributed in conception and design and acquisition of data, analysis and interpretation of data, drafting the manuscript and revising it critically; HG has contributed in conception and design of data, drafting the manuscript and given the final approval of manuscript. Both authors read and approved the final manuscript.
